# Persistent Intracranial Hypertension and Severe Hypoglycorrhachia in an Adult With Giant Congenital Melanocytic Naevi

**DOI:** 10.7759/cureus.51420

**Published:** 2023-12-31

**Authors:** Debbie Kong, Sanihah Abdul Halim

**Affiliations:** 1 Department of Neurosciences, School of Medical Sciences, Universiti Sains Malaysia, Kubang Kerian, MYS; 2 Department of Internal Medicine (Neurology), School of Medical Sciences, Universiti Sains Malaysia, Kubang Kerian, MYS; 3 Brain and Behaviour Cluster, School of Medical Sciences, Hospital Universiti Sains Malaysia, Kubang Kerian, MYS

**Keywords:** csf diversion, neurocutaneous melanosis, hypoglycorrhachia, intracranial hypertension, giant congenital melanocytic naevi

## Abstract

A 29-year-old female, with giant congenital melanocytic naevi (GCMN) presented with a-year history of symptoms and signs of intracranial hypertension. Investigations revealed raised cerebrospinal fluid (CSF) pressure and severe hypoglycorrhachia (low CSF glucose) without pleocytosis. Initial contrast-enhanced brain MRI was normal, but a repeat MRI after a year showed meningeal enhancement with mild communicating hydrocephalus. The raised intracranial pressure was treated with a lumbar-peritoneal shunt. Intraoperative CSF cytology revealed an abundance of squamous epithelia and degenerative cells, but no malignant cells. Her symptoms recovered with CSF diversion via shunt placement, but the hypoglycorrhachia remained. This case highlights the rare occurrence of a non-inflammatory cause of both intracranial hypertension and severe hypoglycorrhachia in a GCMN adult patient, with progressive radiological changes over time, consistent with a diagnosis of neurocutaneous melanosis.

## Introduction

Congenital melanocytic naevus (CMN) is defined as a melanocytic proliferation in the skin that is present at birth. It can reach a large diameter of ≥ 20 cm in adulthood [1]. The majority of patients with giant CMN (GCMN) have somatic NRAS genetic mutation at a frequency of 95% [2]. GCMN tend to develop melanoma or neurocutaneous melanosis (NCM), with two years of age usually being the peak [3]. Rarely, some cases of NCM begin during adulthood, in the second and third decades of life [1,3]. NCM occurs predominantly in association with large sizes or multiple CMN due to the extension of benign or malignant pigment cells along the leptomeninges [1]. Diagnostic criteria and neuroimaging findings of NCM have been published [3]. It is important to recognize that unexplained neurological manifestations such as persistent intracranial hypertension and hypoglycorrhachia can be a part of disease complications in patients with NCM. In our case, the excessive proliferation of benign cells along the meningeal layers could have consumed the glucose in the cerebrospinal fluid (CSF) which explains the hypoglycorrhachia [3]. In GCMN adults presenting with intracranial hypertension or a non-inflammatory type of hypoglycorrhachia, NCM should be suspected as the most likely rare cause. 

## Case presentation

A 29-year-old female, with CMN, presented with recurrent episodes of frontal headache, associated with early morning vomiting and blurred vision for the last year. No fever or constitutional symptoms were reported, as well as no significant drugs, family history, or childhood epilepsy. She did not have a history of contact, or chronic respiratory or bowel symptoms to suggest tuberculosis. No symptoms of connective tissue diseases. She was admitted multiple times to another hospital. She was assessed for intracranial hypertension after recurrent high CSF pressure (30-60 cm CSF), which was associated with a markedly low CSF glucose (0.5-0.9 mmol/L) despite normal serum glucose level on four occasions. CSF protein was slightly elevated (0.45 -0.47g/L) without any leucocytes. CSF investigations for both tuberculosis and viral polymerase chain reaction (PCR), cryptococcal antigen, gram-stain, and cultures were repeatedly negative. She received empirical antibiotic ceftriaxone for 14 days, despite no evidence of leucocytes in the CSF during her first presentation. However, the treatment did not have any impact on the abnormal pressure or glucose content.

**Figure 1:**
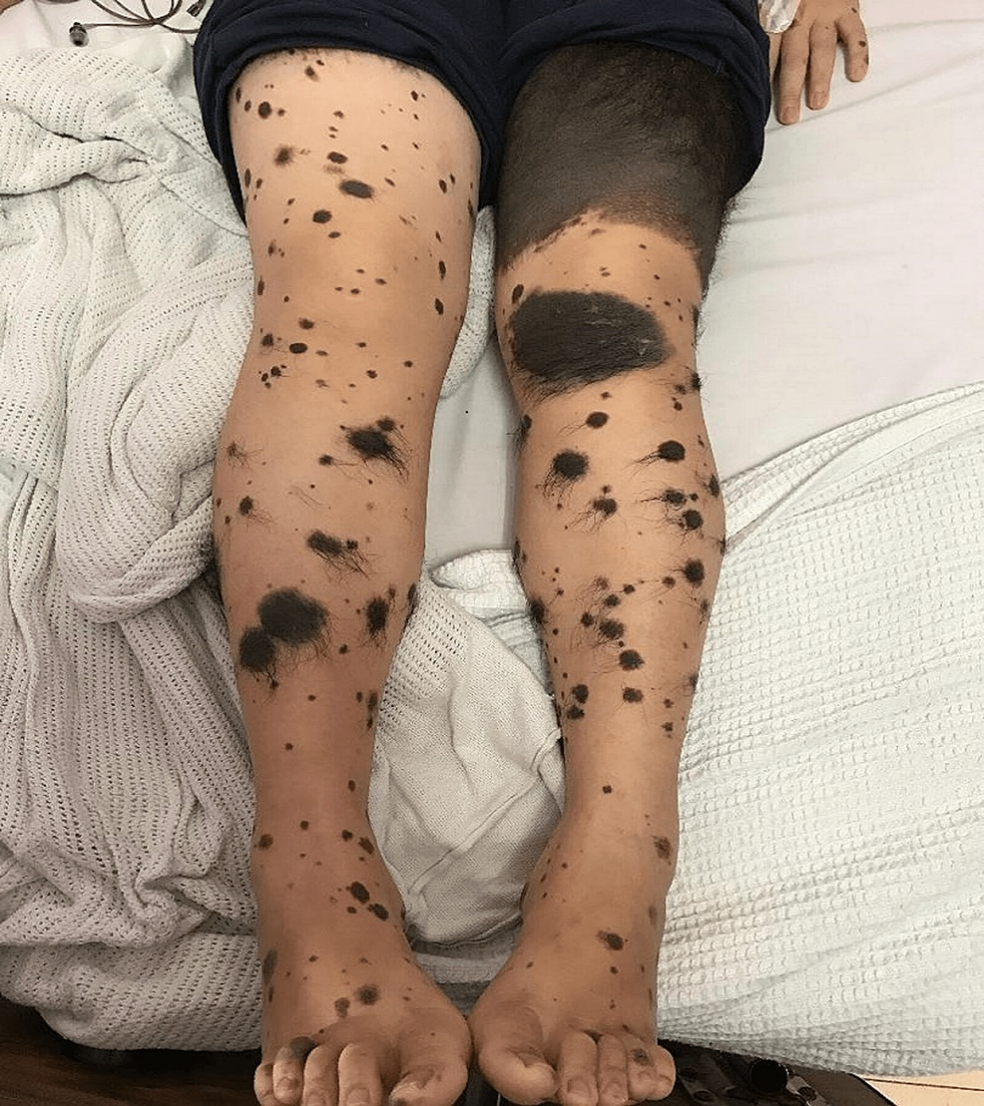
Various sizes of small and giant congenital melanocytic naevi over lower extremities.

Clinical examination revealed scattered, multiple sizes naevi involving 80% of the surface area of her trunk and extremities (Figure 1). She had bilateral papilloedema with a visual acuity of 6/9. She was conscious and afebrile with normal vital signs. No meningism or cranial nerve palsy was noted. Other neurological and systemic examinations were unremarkable.

The initial contrast-enhanced brain MRI was normal. No abnormal meningeal thickening, enhancement, or hydrocephalus were noted. Brain magnetic resonance angiography (MRA) and venography (MRV), as well as whole spine MRI were also normal. Other test results were unremarkable: blood counts, erythrocyte sedimentation rate, c-reactive protein (CRP), human immunodeficiency virus (HIV), Venereal Disease Research Laboratory (VDRL), anti-nuclear antibody, antineutrophil cytoplasmic antibodies (ANCA), complements C3 and C4, tumour markers carcinoembryonic antigen (CEA), cancer antigen 125 (Ca-125) and serum cryptococcal antigen. Chest X-ray and abdominal ultrasound were normal. Tuberculin test was 0 mm. Skin biopsies were taken from two sites and showed multiple compound naevi, without evidence of melanoma.

A presumptive diagnosis of NCM was made based on underlying GCMN and skin biopsy results. A trial of treatment with acetazolamide, to reduce the intracranial pressure was unsuccessful. A second brain MRI was repeated after 6 months, but it did not show much changes as compared to the first brain imaging. A lumbo-peritoneal shunt was inserted in view of persistent symptoms. Intraoperatively, clear CSF was drained. Degenerative cells and abundant normal squamous epithelia were detected in CSF cytology. There was persistent hypoglycorrhachia noted in the CSF.

**Figure 2:**
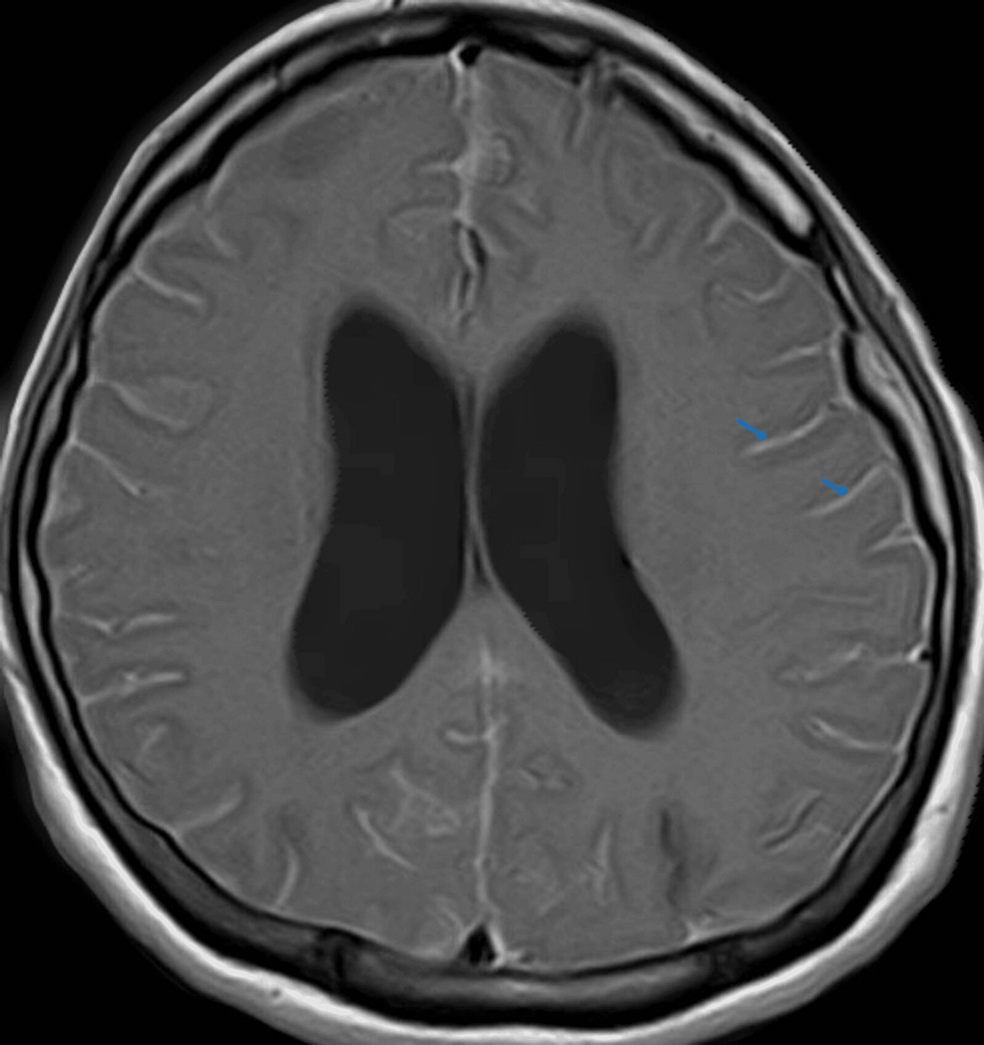
Gadolinium-enhanced T1-weighted brain MRI (axial view) after a year of follow-up shows diffuse leptomeningeal enhancement (blue arrow).

Her headache resolved and the vision improved post-operatively. She had an episode of generalized seizure two months later but responded well to levetiracetam 500 mg twice a day. Electroencephalography (EEG) was performed and showed occasional sharp waves over the right temporal region. A third brain MRI was done 8 months after the second imaging and it showed mild communicating hydrocephalus with diffuse leptomeningeal enhancement (Figure 2). However, there was no symptom of intracranial hypotension to explain the new development of leptomeningeal enhancement. She was stable within a year of follow-up visits.

## Discussion

NCM is a rare disorder [3]. During embryogenesis. neural crest melanoblasts migrate along the leptomeninges to other sites such as skin, mucous membranes, mesentery, eyes and ears, where they differentiate into dendritic melanocytes [4]. Dysregulation during neuroectodermal development of the skin and leptomeninges explains the pathogenesis of GCMN and NCM [4,5]. CMN patients with large-size naevi, multiple satellite lesions, and axial lesions over the posterior head, neck, and paravertebral area are at higher risk of developing NCM [1]. NCM should be suspected in any patient with high-risk features presenting with neurological symptoms such as headache due to intracranial hypertension, seizures, hydrocephalus, cranial nerve palsy, weakness, or neuropsychiatric manifestation. GCMN can be associated with other structural central nervous system malformations: arachnoid cysts, choroid plexus papilloma, cerebellar astrocytoma, spinal dysraphism, and type I Arnold-Chiari malformation [1,4,5].

In this case, a presumptive diagnosis of NCM was made based on underlying GCMN and skin biopsy results. However, the typical MRI features of NCM such as hyperintense areas in T1-weighted imaging as well as leptomeningeal thickening or enhancement, were not visualized in the first brain MRI [4]. Chronic meningitis is an important diagnosis that must be considered in cases with persistent symptomatic intracranial hypertension and a markedly low CSF glucose (hypoglycorrhachia) [6]. However, chronic infections such as tuberculosis or fungal were excluded due to the absence of fever or meningism, negative serum or CSF infectious and inflammatory markers, and negative tuberculin test. She had no symptoms and signs to suggest systemic malignancy. Furthermore, malignant meningeal deposits were not identified from the MRI and CSF results. Brain and spine MRI ruled out other causes of raised intracranial pressure: venous sinus thrombosis, vasculitis, hydrocephalus, intra-cranial or intra-spinal mass, or any structural lesions [6]. Idiopathic intracranial hypertension (IIH) is one of the differential diagnoses but was excluded by an abnormal CSF content [7].

In this case, the primary concern was persistent symptomatic intracranial hypertension and a markedly low CSF glucose (hypoglycorrhachia). Intracranial hypertension can be caused by a few mechanisms that disturb the regulation of intracranial pressure: intraparenchymal or mass lesion, vascular or cerebral circulation disorder, or impairment in CSF dynamics and idiopathic intracranial hypertension [6]. Neuroimaging that was performed in this patient excluded intraparenchymal brain lesions and vascular disorders. The mechanism of intracranial hypertension can be due to a disorder of CSF dynamics: overproduction of CSF, impairment of CSF flow, or reduction in CSF absorption [6]. Among these mechanisms, impairment of both CSF outflow and absorption was suspected.

The CSF pressure was markedly high, but there was no ventricular enlargement or hydrocephalus noted in the first two brain MRIs, which were done within 1-1.5 years of the initial symptoms. This could be explained by an intracranial compensatory mechanism by cerebral autoregulation in chronic elevation of intracranial pressure (ICP), which is almost similar to the mechanism of increased ICP in idiopathic intracranial hypertension (IIH): whereby the ventricles are not dilated even with very high CSF pressure [6,7].

In progressive NCM, involvement of leptomeninges and arachnoid granulation may lead to the chronic reduction of CSF absorption [8,9]. Excessive benign melanotic cells along the meninges may be present in multiple areas that may have macroscopic pigmentation under normal circumstances: the brain convexities, the base of the brain, ventral surfaces of the pons, medulla and upper cervical and lumbosacral spinal cord [4,5]. It can be nodular or diffuse. Although meningeal biopsy was not performed in this case, and no melanocytes were seen in the CSF, the detection of degenerative cells with an abundance of normal squamous epithelia from the intraoperative CSF cytology suggests excessive proliferation of benign cells along the meningeal layers, with features similar to skin structures. Acosta et al. reported a child with GCMN presenting as acute hydrocephalus due to neurocutaneous melanosis [9]. Melanocytes were absent in the CSF cytology despite brain MRI evidence of melanocyte accumulation within the hippocampi, medulla, and cerebellum [9]. In our case, we postulated that the abundance of benign cells could have consumed the glucose in the CSF which explains the hypoglycorrhachia [8]. The absence of leucocytes, with borderline normal CSF protein, indicates that an inflammatory process is not necessarily the cause of low CSF glucose [8]. Impaired CSF glucose transport is another possibility that must be considered. Yet, the rise in CSF pressure could not be explained by this theory alone.

The diagnostic criteria for NCM have been revised and proposed by Kadonaga and Frieden [3]. The diagnosis can be made by either: (a) the presence of large (≥ 20cm in adult) or multiple (three or more) CMN, associated with either meningeal melanosis or melanomas, (b) the absence of cutaneous melanoma, except in patients in whom meningeal lesions are histologically benign and (c) absence of meningeal melanoma, except in patients with benign cutaneous lesions on histological examination [3]. A definite diagnosis can be made only by histologic confirmation. Without a biopsy, all others are considered provisional diagnoses.

Typical MRI features of NCM in adult patients are hyperintensities on T1 weighted imaging, or diffuse thickening and contrast enhancement of the leptomeninges or hydrocephalus [8]. Rarely, intraparenchymal mass without leptomeningeal involvement may be present [10]. NCM may not be evident radiologically in some patients. Symptomatic patients with neurological manifestations but normal neuroimaging have been reported [4,11]. In a study by Ruiz-Maldonado et al., 11 out of 13 patients with GCMN of the head and neck with neurological features had no evidence of NCM in their brain CT or MRI [11].

Our patient had a slow progression of disease which was symptomatic only during adulthood, at the age of 28 years old. Despite an unremarkable finding in the first two brain MRIs and the absence of cutaneous melanoma, the provisional diagnosis of NCM was made, in view of she had increasing size and number of GCMN with high-risk features of CMN over the years, and extensive investigations to look for common causes of intracranial hypertension and hypoglycorrhachia turned out to be negative [3,8]. The typical MRI features of NCM were captured only in the third MRI, with diffuse leptomeningeal involvement and mild hydrocephalus indicating a progressive disease. Increased opening pressure during lumbar puncture, elevated CSF protein and low glucose levels had been found in adult patients with NCM [8]. A meningeal biopsy to determine the presence of melanocytic cells along the meningeal layers and genetic evaluation would be extremely helpful in confirming the diagnosis of NCM according to the current diagnostic criteria [3].

Up to date, there is no effective treatment to modify the disease course. Treatment aims to reduce symptoms and neurological complications, primarily by surgical intervention in a large intracranial lesion or CSF diversion via shunt placement for intracranial hypertension [12,13]. Radiotherapy and chemotherapy have been offered in severe disease but with limited benefit. The overall prognosis is poor in diffuse leptomeningeal involvement, with less than 3 years survival [3,13,14].

## Conclusions

Neurocutaneous melanosis (NCM) rarely begins during adulthood and diagnosis may be delayed due to late presentation. Persistent intracranial hypertension and hypoglycorrhachia in giant congenital melanocytic naevi patients should raise suspicion of neurocutaneous melanosis. Typical neuroimaging findings of NCM may not be present in the early course of disease, despite abnormal neurological symptoms. There is no specific therapy for NCM. Treatment is aimed to reduce symptom and neurological complications.
